# Nutritional Intake in Venovenous ECMO Patients: A Single-Center Study in a North American PICU

**DOI:** 10.3390/nu16223813

**Published:** 2024-11-07

**Authors:** Annika Lintvedt, Isabella Purosky, Benjamin Kogelschatz, Megan Brackmann, Erika Heinze, Jessica Parker, Brooke Dudick, Jamie Mcdiarmid, Elizabeth Rosner, Brian Boville, Mara L. Leimanis-Laurens

**Affiliations:** 1 Department of Pediatrics and Human Development, College of Human Medicine, Michigan State University, Grand Rapids, MI 49503, USA; lintvedt@msu.edu (A.L.); puroskyi@msu.edu (I.P.); brackma8@msu.edu (M.B.); elizabeth.rosner@helendevoschildrens.org (E.R.); brian.boville@helendevoschildrens.org (B.B.); 2 Pediatric Critical Care Medicine, Helen DeVos Children’s Hospital, Grand Rapids, MI 49503, USA; jamie.mcdiarmid@corewellhealth.org; 3 Corewell Health, Grand Rapids, MI 49503, USA; erikaheinze58@gmail.com (E.H.); jessica.parker2@corewellhealth.org (J.P.); brooke.dudick@corewellhealth.org (B.D.)

**Keywords:** extracorporeal membrane oxygenation, pediatric intensive care unit, venovenous, venarterial, nutritional support, delay and interruption of nutritional support, vasoactive inotrope score, pediatric nutrition, critical care, feeding tolerance

## Abstract

Background/Objectives: Multiple independent variables were analyzed to determine total nutritional intake (caloric and protein), while reviewing vasoactive inotrope scores (VISs). Methods: Retrospective data were collected for nutritional intake (N = 64), daily VISs, extracorporeal membrane oxygenation (ECMO) complications, delays in nutritional intake (>48 h), reason for delay, and interruptions in nutrition support. Results: VISs and ECMO characteristics analyzed by box–whisker plots demonstrated that ECMO complications prior to 24 h, cardiac arrest 24 h prior to ECMO, pediatric ECMO patients, venoarterial ECMO type, having a cardiac ECMO indication, and ECMO centrifugal pump had higher VISs. A regression analysis revealed that venovenous ECMO patients and the centrifugal pump type had higher caloric and protein intake; subsequently, for each increase in VIS, caloric intake decreased by −0.54. Sixteen patients did not receive nutritional support while on ECMO (9/16; 56% cardiac); 12/48 (25%) had a delay, with the cardiac patients nearing statistical significance. Conclusion: Venovenous ECMO (non-cardiac) correlated with higher nutritional intake. The enteral administration of nutrition had a lower VIS on average compared to the other three groupings, namely enteral vs. parenteral; enteral vs. both enteral and parenteral; enteral vs. no nutrition. This study provides additional clinical insight on nutritional intake in ECMO patients.

## 1. Introduction

Extracorporeal membrane oxygenation (ECMO) support is a modified form of cardiopulmonary bypass, and its use continues to increase within neonatal, pediatric, and adult intensive care units as a potentially life-saving modality. Venovenous (VV) ECMO is for lung support, whereas venoartierial (VA) ECMO supports both the lungs and the heart. This patient population is the most acutely ill in the pediatric intensive care unit (PICU), and nutrition is impacted by many independent variables and risk factors. While decision making within the PICU often follows standardized guidelines, guidance for nutrition support is more flexible and less outlined. Dietitians frequently staff patient-centered rounds in the PICU, providing insight on nutrition support initiation, nutrition support type, calorie adequacy, and more. The decisions to delay, interrupt, or cease nutrition support can happen at any point throughout a PICU stay, and could have implications in individual morbidity and mortality [1,2].

Vasoactive inotropic scores (VISs) are a calculated value that determines the amount of vasoactive support that a patient is utilizing (such as epinephrine, norepinephrine, dopamine, milrinone, dobutamine, and vasopressin). Some studies utilize VISs as a determination of vasoactive support, while others utilize them as surrogates for illness acuity, hazard of mortality, or degree of hemodynamic compromise [3,4,5,6]. VISs together with the clinical outcomes of feeding intolerance, facilitate a comparison that elucidates risks for bowel ischemia [6]. For this work, we reported VISs as they affected the various sub-groups of patients and outcomes, such as total caloric and protein intake. These results were absent from our prior publication on this patient cohort [7].

Significant delays and interruptions of nutritional support have been noted, particularly amongst critically ill patients [8,9]. The administration of nutrition is complex and reliant on specific patient characteristics, including but not limited to necessity for fluid restriction, mechanical access issues, and multiple scheduled and/or emergent procedures [10]. Despite its proven importance, previous work has indicated that patients who are receiving ECMO struggle to meet their daily caloric requirements. In our most recent cohort study, it was found that none of the patients met their caloric and protein requirements while receiving ECMO support [7].

The 2020–2030 Strategic Plan for Nutrition Research is attempting to enhance ongoing research efforts across the National Institute of Health (NIH) to improve health and prevent or combat disease affected by nutrition [11]. The importance of nutrition in in-patient settings is amplified by the severity of disease burden, and precision medicine is key to improving outcomes. Thus, the NIH initiative to improve nutrition research is paramount in this patient population, yet significant knowledge gaps remain. Nutritional pediatric randomized control trials in ECMO are limited by a low incidence, ethics, and limited funding. Taken from Mehta et al. (2017): “Optimal protein intake and its correlation with clinical outcomes are areas of great interest. The optimal route and timing of nutrient delivery are areas of intense debate and investigations” [9,12,13]. The purpose of this case series was to delve into specific patient characteristics related to delays and interruptions in nutrition support and to investigate gaps in understanding regarding various independent variables. From previous work looking at a cohort of eight ECMO patients, we had reported that cardiac patients received lower amounts of total calories as compared to non-cardiac ECMO patients [14], prompting further evaluation. The work described is clinically relevant to clinicians, cardiologists, nurse practitioners, dietitians, nutritionists, pediatric intensivists, and ECMO technicians.

## 2. Materials and Methods

### 2.1. Nutritional Assessment

Every effort is made to initiate nutrition support within 24–48 h of admission in both the PICU and the Pediatric Cardiac Intensive Care Unit (PCICU), as guided by The Provision and Assessment of Nutrition Support Therapy in the Pediatric Critically Ill Patient: Society of Critical Care Medicine (SCCM) and American Society for Parenteral and Enteral Nutrition (ASPEN) [9]. The decision to initiate nutrition support (parenteral or enteral nutrition) is discussed daily in multidisciplinary rounds. At the authors’ institution, the PICU rounding team typically includes the intensivist, fellow, residents, nurse practitioners, physician assistants, bedside nurse, pharmacist, and dietitian. Similarly, the PCICU rounding team is compromised of the same roles, with the addition of the cardiothoracic surgeons and a cardiologist.

### 2.2. Decision Support

In the PICU, the intensivist typically will make the final decision on the initiation of nutrition support. In the PCICU, the decision is more of a collective decision between the intensivist, cardiothoracic surgeon, and cardiologist. Decisions are made based on a variety of factors, including, but not limited to, hemodynamic stability, gastrointestinal complications, device access, need for further procedures, and fluid restriction. Once nutrition support is initiated, it continues to be discussed daily during rounds. The bedside nurse will regularly assess enteral feeding tolerance. If intolerance is present, they will hold feedings and discuss further plan with either the mid-level or attending. Any other factors that require holding either enteral or parenteral nutrition are determined by the intensivist, cardiothoracic surgeon, cardiologist, nurse practitioner, or physician assistant.

### 2.3. Feeding Algorithm

A feeding algorithm is utilized in the PICU that includes guidelines for advancement and the holding of enteral feedings pending an evaluation of tolerance. While the guide is typically followed for feeding advancement, the length of time for holding feedings due to intolerance may not always be followed per the guide’s suggestion. Similarly, in the PCICU, low-risk and high-risk feeding advancement guidelines are followed. However, at this time, the PCICU does not utilize a feeding algorithm that includes guidelines for holding of enteral feedings based on intolerance and is based on provider discretion.

### 2.4. Study Design

#### 2.4.1. Site, Patient Population

A consecutive series of patients were included over a four-year time period, as previously reported [7]. Data collection was at Helen DeVos Children’s Hospital (HDVCH), located in Grand Rapids, MI. Helen DeVos Children’s Hospital falls under the umbrella of Corewell Health Hospital System, which consists of 3 hospitals within Grand Rapids and 8 regional hospitals across the state. HDVCH is a tertiary care referral center in West Michigan. HDVCH PICU has over 1500 admissions per year with over 6000 patient days. Twenty-four board certified intensivists cover a 24-bed unit with capability to care for up to 36 critically ill children.

#### 2.4.2. Data Collection for Delays and Interruptions of Nutritional Support

A retrospective chart review of patients (N = 64) requiring ECMO at HDVCH between 2018 and 2022 was evaluated as previously described [7]. Variables included demographics, outcome data including daily enteral and parenteral nutrition data, laboratory values, ECMO complications, and delays in initiation of nutritional intake (>48 h), including the reason for delay and the presence of interruptions in nutrition support.

The extent of delay was defined by 48–60 h, 60–72 h, and >72 h after ECMO initiation. The reasons for delay were categorized as clinical status, procedures, no access, or mechanical complications, and planned extubation.

Interruptions were defined as the cessation of nutrition support noted within chart review and total number of interruptions during ECMO was listed. VISs were calculated after collecting dosages of dopamine, dobutamine, epinephrine, norepinephrine, milrinone, and vasopressin being given closest to 0600 each day.

Lastly, PRISM scores were calculated utilizing the CCPRN tool, which included age and other variables such as systolic blood pressure, temperature, heart rate, blood gasses, electrolytes, and blood cell abnormalities, as previously reported [7]. Vital signs and laboratory values were taken closest to the time of PICU admission, no more than 24 h prior or 24 h after admission. Data were reviewed in EPIC and entered in REDCap^®^ (14.5.10 (2024 Vanderbilt)), a secure data management system.

#### 2.4.3. Data Analysis

Numeric data were expressed as mean ± standard deviation or median (25th, 75th percentile) depending on normality of the data. Categorical data were expressed as frequency (%). For numeric group comparisons, independent *t*-tests were used, or a Wilcoxon Rank Sum, depending on normality. For numeric group comparisons with more than two variables (i.e., pediatric cardiac, pediatric non-cardiac, vs. neonate) with numeric outcomes, One-Way ANOVA or Kruskal–Wallis was utilized (which is a non-parametric version of One-Way ANOVA), depending on normality. If the outcome was categorical, a Chi-Square or Fisher’s Exact Test was used, depending on expected cell counts. All analyses were assessed at an alpha of 0.05. Delays in nutritional intake were reviewed and analyzed as categorical and continuous variables.

Linear regression analysis was completed for both total calories, and total protein intake and all six of the independent variables of interest were assessed (ECMO complications: Yes vs. No; ECMO indication: cardiac vs. non-; age group: neonatal vs. pediatric; ECMO type: VA vs. VV; ECMO pump type: centrifugal vs. roller), including the VISs in the initial model, using backward stepwise elimination to exclude those independent variables that were not significant, i.e., variables were placed in the model and then removed one by one based on the highest *p*-value.

#### 2.4.4. Quality Control

A randomized quality control was performed to ensure accurate data collection. Two investigators randomly selected record numbers and reviewed the individual chart and data entry for accuracy. A total of 5% of the patients were re-reviewed in the initial quality control process. A score of one or zero was assigned to the five questions for each patient depending on whether the two raters came to the same conclusion. Edits were made based on the results of the quality control and were then re-assessed for adequacy. The initial inter-rater reliability was approximately 81% (13/16 points achieved). Members of the study team determined the discrepancy, and the data were corrected accordingly. The discrepancy was regarding the specific definition of what was considered a feed interruption. This allowed the study team to develop a more granular definition of an interruption, and it was determined that greater than or equal to 8 h would be considered the cut-off. Subsequently, all patient data were edited to reflect this change, which corrected the initial inter-reliability percentage to 100% (16/16 points achieved).

## 3. Results

### 3.1. Patient Summaries

#### Clinical Flow Chart for Delay of Nutritional Support

**Figure 1:**
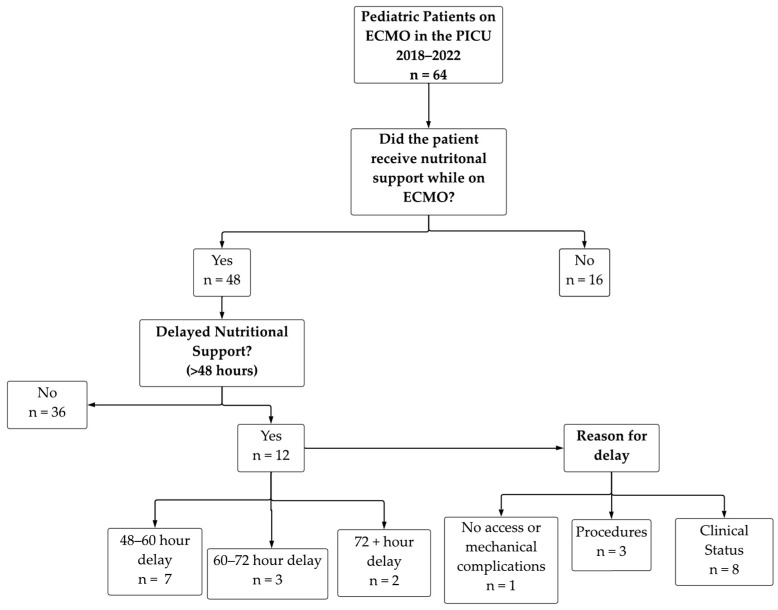
Patient flow chart (N = 64) over 467 ECMO days.

A total of 64 patients were included for initial chart review to determine a delay or interruption of nutritional intake during their ECMO course (Figure 1), as presented later in the report (see Section 3.3). Sixteen patients were excluded from further analysis, as they did not receive any nutritional intake during their ECMO course (see Table 1). From the remaining 48 patients, 12 experienced delays in nutritional support (48–60 h; *n* = 3 60–72 h; *n* = 2 > 72 h), which included 8 due to clinical status, 3 due to procedures, and 1 due to no access or mechanical complications. This flow chart illustrates the subsequent patient groupings that will be described in later tables and figures.

### 3.2. Summary of Vasoactive Inotrope Scores

#### 3.2.1. Total Cohort Iontrope Values over 467 ECMO Days

**Figure 2:**
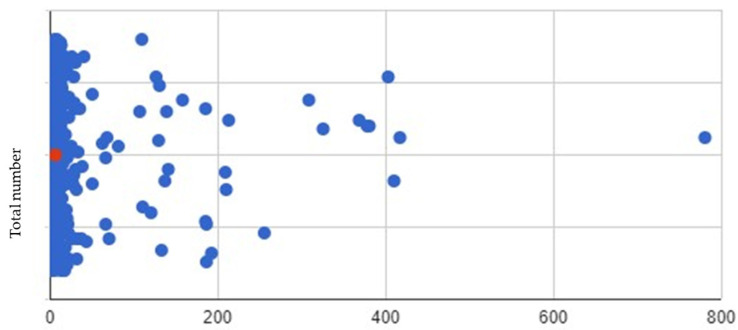
A scatterplot of PICU vasoactive inotrope scores (*n* = 583) over 467 ECMO days. The red dot is the median value, which is zero; “total number” *Y* axis, and “vis score” *X* axis.

VISs were reviewed (*n* = 581) and extracted for each patient over their ECMO course over 467 days. A scatterplot of the total VISs is visualized in Figure 2 (additional scatterplots for all six inotropes are available for review in Supplementary Figure S1). A further breakdown of the individual inotrope values and total sums of dopamine (244 mcg/kg/min), dobutamine (2 mcg/kg/min), epinephrine (20.5 mcg/kg/min), milrinone (167.29 mcg/kg/min), vasopressin (0.67 U/kg/min), norepinephrine (8.04 mcg/kg/min), total VIS are summarized in Table 2. Missing data were <1% for inotropes reviewed. The top three mean values of drugs included dopamine (0.42), milrinone (0.29), and epinephrine (0.04). Raw values and heatmap distribution of drug values was further provided (Supplemental Table S1). These data illustrate the degree of variability in VISs. From this point on, we sought to breakdown the VISs based on independent variables.

#### 3.2.2. Vasoactive Inotrope Scores by ECMO Characteristics

**Figure 3:**
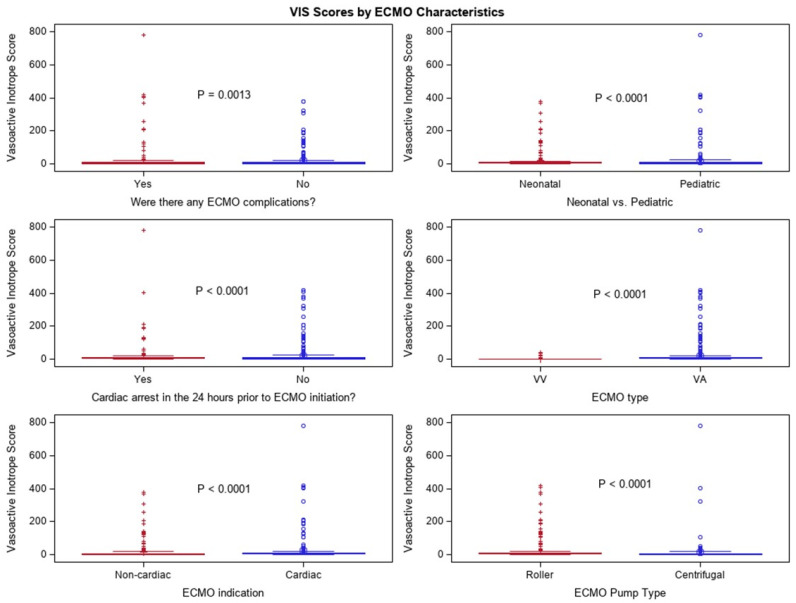
Box–whisker plot of six ECMO sub-groupings.

We had several sub-group analyses, as one goal of this investigation was to identify any independent variable that might be most affected by VISs, therefore influencing subsequent nutritional intake in a later analysis. We stratified the following groupings as binary (yes/no): ECMO complication in the prior 24 h; cardiac arrest in the 24 h prior to ECMO initiation, and as categorical for neonatal vs. pediatric; ECMO type (VV vs. VA) ECMO; ECMO indication (non-cardiac vs. cardiac), and ECMO pump type (roller vs. centrifugal). Box–whisker plots are presented, where the y-axis is the VIS and there are two comparative groups on the x-axis. The results demonstrate significant *p*-values for “yes” to ECMO complication in the prior 24 h (*p* = 0.0013), and cardiac arrest in the 24 h prior to ECMO initiation (*p* ≤ 0.0001). The following were all significant (*p* ≤ 0.0001): pediatric ECMO patients, VA ECMO type; cardiac ECMO indication, and ECMO centrifugal pump type (Figure 3; Supplementary Table S2). It is likely that the ECMO centrifugal pump type is confounded by size, since centrifugal pumps are used on infants over 30 days and 10 kg, which has previously been associated with higher rates of complications in neonate populations [15,16]. Additional questions surrounding the administration route of the nutrition are addressed in the following paragraph.

Surprisingly, when we ran correlations with VIS and total calories and total protein, there was no correlation the correlation coefficients were less than 0.15. When looking at the median VISs and total nutritional intake via the route of administration, significance was found between the groups enteral (*n* = 130; 0 [0, 5]), parenteral (*n* = 250; 8 [5, 11]), both enteral and parenteral (*n* = 62; 8 [5, 8]), and no nutrition (*n* = 142; 10 [3, 14]), with an overall *p*-value of <0.0001. Looking at the pairwise comparisons, enteral had a lower VIS on average compared to the other three feed type groups, namely enteral vs. parenteral; vs. both; and vs. no nutrition, (all *p*-values ≤ 0.0001).

#### 3.2.3. ECMO Characteristics, Linear Regression and Caloric and Protein Intake

In order to determine independent variables for the influence of the nutritional intake of pediatric ECMO patients, we pursued a linear regression analysis. Those with a roller pump type have lower caloric intake by −305.33 compared to those with a centrifugal pump, while holding all other variables constant (*p* < 0.0001). We are 95% confident that the caloric intake difference between the two groups lies between −383.04 and −227.63. Those with VV ECMO had a higher caloric intake, by 422.07 compared to those with VA, while holding all other variables constant (*p* < 0.0001). For each increase in VIS, caloric intake decreased by −0.54 (*p* = 0.0150). A linear regression analysis of variation demonstrated that the model as a whole was statistically significant (*p* ≤ 0.0001), the R-square was found to be 0.4137, which implies that 41% of the variation in caloric intake can be explained by the pump type, ECMO type, and VIS (Table 3). There is a moderate association between pump type, ECMO type, and VIS.

Those with a roller pump type have a lower protein intake, by −24.47 compared to those with centrifugal pump, while holding all other variables constant (*p* < 0.0001). Those with complications have a higher protein intake, by 6.18 compared to those without complications, while holding all other variables constant (*p* = 0.0030). Those with an ECMO type of VV have higher protein intake, by 33.01 compared to those with VA, while holding all other variables constant (*p* < 0.0001). We are 95% confident that the protein intake difference between the two groups lies between 26.05 and 39.97. This shows the model as a whole is statistically significant. The predictors can reliably predict total protein intake, and the R-square is 0.4253 (Table 4).

#### 3.2.4. ECMO Predictors Using Linear Regression for Caloric and Protein Intake

**Figure 4:**
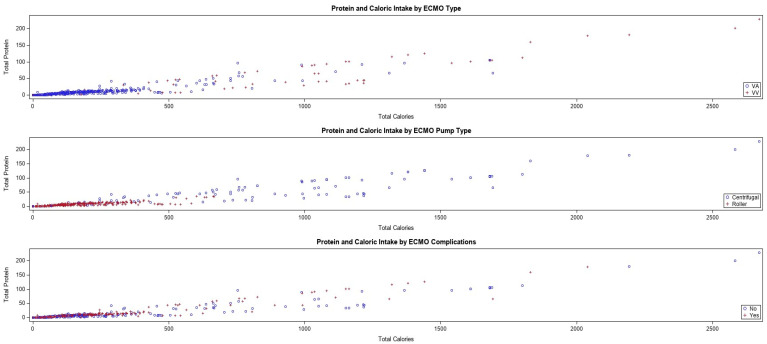
Protein and caloric intake by ECMO type (VA vs. VV); ECMO pump type (centrifugal vs. roller); ECMO complications in the 24 h prior to ECMO initiation.

Furthermore, we wanted to explore the predictive likelihood of independent variables for nutritional intake in ECMO patients. A scatterplot analysis was run to determine which of the independent variables as previously described influenced the caloric and protein intake. As described in the Materials and Methods section, a linear regression analysis was completed for both total calories, and total protein intake and all six of the independent variables of interest were assessed (ECMO complications: Yes vs. No; ECMO indication: cardiac vs. non-; age group: neonatal vs. pediatric; ECMO type: VA vs. VV; ECMO pump type: centrifugal vs. roller), including the VISs in the initial model, using backward stepwise elimination to exclude those independent variables that were not significant (Figure 4).

The predictors were reliably able to predict total calorie intake. From this, we were able to determine that ECMO type, ECMO pump type, and ECMO complications in the prior 24 h were correlated with total nutritional intake. VA ECMO is used for heart and lung support, whereby VV is only used for lung support. From previous results, we found that centrifugal pump had higher VISs, and has been associated with higher rates of major complications [17]. Additional correlation analyses were run for VIS vs. calories and protein by independent variables (see Supplementary Table S3).

#### 3.2.5. Descriptive Analysis of Patients on ECMO with No Nutritional Support

Outstanding from the previous manuscript describing this patient cohort with no nutritional support during their ECMO course, a detailed description was summarized (Table 1). Patients that were further excluded from the analysis (*n* = 16) did not receive any nutritional support during their ECMO course (Table 1). It was debated as a study team whether these patients should be included as the “extended delay/interruption” of nutritional support for the cohort; however, ultimately, it was decided to separate these patients and not include them in further analyses. The results, however, were tabulated as a sub-set as a case series, summarizing their clinical course according to the following variables: primary, secondary, tertiary diagnosis, age in years (0–2; *n* = 11; >2–6; *n* = 1; 6–12; *n* = 0; >12; *n* = 2); gender (male: 9/16; 56%), ECMO time in hours (<24; *n* = 7; 24–48; *n* = 2; >48–72; *n* = 3; >72–96; *n* = 1; >96; *n* = 2); ECMO indication was cardiac in 9/16 (56%). For patients on ECMO < 24 h, six out of seven resulted in a mortality. Primary diagnosis varied from infectious agents to congenital heart issues. Reasons for delay included (not exclusively): fluid status (overload, or restriction) and gastrointestinal issues (bleed or intolerance), and were within a less than 48 h timeframe.

### 3.3. Trajectories of Nutritional Support

#### 3.3.1. Interruption of Nutritional Support

Patients were further reviewed for either delay in nutritional support (Figure 1), or an interruption of nutritional support (Table 5). Most patients experienced a total of one interruption (11/12; 91.7%), with one patient reporting six interruptions (1/12; 8.3%). Additional analysis was carried out on the interruption of nutritional support group, in which 17 patients were removed. No statistically significant findings were reported for the following sub-groupings, as outlined above: neonatal vs. pediatric; ECMO type (VA vs. VV); ECMO pump type (centrifugal vs. roller); ECMO indication (cardiac vs. non-cardiac); ECMO complication in the prior 24 h (Yes vs. No), and median VIS (Supplementary Table S4).

#### 3.3.2. Delay of Nutritional Support by Categories

A total of 12 patients experienced a delay in nutritional support from the 48 (25%) reviewed (Table 6). A delay in nutritional support was analyzed according to the patient sub-grouping, as previously listed. Although none reached statistical significance, the cardiac sub-grouping neared statistical significance (*p* = 0.0665). This finding is supported by the previous result, whereby the VA ECMO patients had higher VISs, recalling that this ECMO type is for both heart and lung support. The sample sizes were rather small for further analytical stratification; however, we will continue to add to this registry moving forward and anticipate further reports at a later time.

## 4. Discussion

ECMO is a life-saving modality, with a high mortality rate in pediatric populations [18]. We have previously shown that ECMO patients reached higher caloric and protein goals compared to patients with multi-organ dysfunction syndrome (MODS) over the course of an 8-day PICU admission [14]. In spite of the comparative differences initially reported between ECMO and MODS patients, ECMO patients were found to not be meeting their nutritional goals in a subsequent follow-up, retrospective cohort examining 64 patients [7]. This work continues to analyze this patient cohort to identify specific independent variables affecting these nutritional outcomes.

One of the areas of further review included VISs. In a study performed in 2017, VISs predicted an increased mortality hazard to the end of ECMO support, but notably, in multivariable analysis research carried out within the same study, ECMO support and VISs were not associated with mortality independently [5]. The data within this study are comparable to similar data in other groups’ findings that patients who require vasoactive infusions with ECMO are less likely to survive to discharge. It was noted that the use of vasoactive support was an important consideration in initiating enteral nutrition, which could be a reason for this determination [5]. In our cohort, nutritional intake guidelines did not specifically focus on vasoactive infusions but were more based on a variety of factors that may or may not involve vasoactive infusions within a multi-team discussion during rounds on each patient.

In a survey conducted in 2015, it was determined that a patient’s underlying diagnosis and vasopressor support were important factors for physicians to make the decision to start enteral nutrition specifically, although no VIS thresholds have been determined for enteral nutrition implementation [5,19]. In a more recent study in 2022, this threshold was closer to being determined. When reviewing the entire cohort in the study, VISs on the day of initiating enteral nutrition were lower for survivors vs non-survivors, suggesting the possibility of determining a threshold with further research. Higher survival rates were found in situations of early initiation of enteral nutrition in children on ECMO with lower VISs. Patients who had higher VISs were more likely to have gastrointestinal complications such as bleeding [6]. We determined that there was a significant correlation between higher VISs and ECMO indications and complications. Specifically, we focused on the cardiac patients and how this specific indication was tied to higher VISs.

In a similar study to the one currently presented, comparing nutritionally supported vs. no nutrition, the fed group received less vasoactive agent support than the non-fed group, but the difference was only significant on day 1 of nutritional intake. Patients from the cardiovascular group who received vasoactive agents were more likely fed than not [4]. This differs from our study, with the cardiac group tending to be more likely to be unfed, which emphasizes how nutrition initiation is based heavily upon provider discretion.

Literature highlighted mainly the arguments of enteral feeding protocols regarding VISs, but none pertaining to total parenteral nutrition (TPN) as we utilize alongside enteral nutrition in our study. This highlights a deficit and requires further research to assess how TPN fits in with VISs and how they are related, if at all. One study trial compared TPN with enteral nutrition in critically ill patients in shock who received vasopressor support and determined that the mortality did not differ between the two groups [20]. While they did not utilize VISs specifically, they did utilize a necessary comparison between TPN and enteral nutrition while undergoing vasoactive therapy, which provides relevant information for determining the utilization of TPN or enteral nutrition. In their research, there was no difference determined with mortality or hospital stay lengths. While most identify that numerous vasopressor agents in critically ill patients receiving enteral nutrition are safe, further research and continued monitoring needs to be carried out to ensure that the vasoconstrictive effects of vasopressors are not impeding patient safety [21].

Studies in the adult population have indicated that early enteral nutrition support improves outcomes. However, results have not been as convincing in the pediatric population, potentially due to drastic differences in nutrition status prior to ICU admission as well as potential metabolic differences in pediatric patients [7].

Much of the current research has focused on early versus late initiation of nutrition in critically ill patients and its effects on different outcomes. There has been little focus on the frequency of nutrition interruptions. These interruptions may be more impactful on critically ill children, given their higher metabolic rates [22,23]. The patients in this cohort who had cardiac indications for ECMO had poorer nutritional intake when compared to patients receiving ECMO for non-cardiac indications. There is minimal literature surrounding specifics on nutrition delivery (including delays and interruptions) in the cardiac ECMO patient population. This identifies a deficit in knowledge regarding optimal nutrition support in this vulnerable patient population.

Future work could look at any association between nutrition and the initiation of continuous renal replacement therapy (CRRT) while on ECMO [24,25], since TPN is not initiated due to fluid status and fluid overload. However, while on ECMO, CRRT circuit may be added to be able to reduce volume and the initiation of nutritional support, as was speculated by Zhang et al. in a recent review [26]. A deeper investigation could reveal whether those with CRRT received nutrition earlier or in greater amounts in their ECMO run. In a larger sample size, we could stratify patients by severity and determine a predictive cutoff value for routes of administration or predictive nutritional intake. For this, we would need both validation and test populations. Gut dysbiosis and the gut microbiome (as previously examined by our group in infant bronchiolitis populations; Russell et al. [27]) could provide additional insight into diversity and abundance of healthy gut microbes, as it is known that a healthy gut microbiome promotes digestion and absorption, which might be compromised during the hyperoxia caused by ECMO. Recent reviews have started to explore the subject in more depth [28].

Recommendations for clinical practice based on our work include special consideration for VA ECMO populations, which have greater delays in nutritional administration, and less total intake for both protein and calories. Furthermore, a lower VIS facilitates enteral route of feeds; however, a specific cut-off point is not currently suggested from this report.

Limitations for this work include provider bias, as clinical teams change daily, and along with this comes experience levels, years of practice, and the consideration and integration of nutritional practices into a high-volume tertiary care unit, among other factors. Sample size could be augmented with the use of multi-site data; however, nutritional information is not captured as a unique variable in the EMR, and therefore requires manual data extraction. Nutritional data are not included in Virtual PICU Systems, LLC^®^, or ELSO databases as total caloric and protein intake. Additional considerations for future work need to consider nutritional intolerance, malnutrition, micronutrient deficiency, post-PICU syndrome [29], all to be considered in a post-PICU setting, i.e., once the patients leave the PICU, leave the hospital, or continue to rehabilitate in in-patient or outpatient settings. Follow-up data are not included in this current report, nor are non-ECMO PICU days. Prokinetics have been shown to reduce feeding intolerances [30]; however, they were not reported in this study.

## 5. Conclusions

From this detailed case series and descriptive cohort review, we determined that patients from 2018 to 2022 on ECMO experience delays in their nutritional support. Patients who did not obtain nutritional support in the first 24 h of ECMO had a high mortality rate; however, this may have been an incidental finding, as these may have also been the most acutely ill. VISs were inversely associated with caloric intake. Multiple independent variables revealed to be statistically significant in the total calories and protein, namely VV ECMO patients, and higher VISs. Enteral had a lower VIS on average compared to the other three feed type groups, namely enteral vs. parenteral; vs. both; vs. no nutrition. Of particular attention is the PCICU populations, which do not utilize a feeding algorithm that includes guidelines for the holding of enteral feedings based on intolerance; however, this is based on provider discretion.

## Figures and Tables

**Table 1:** Patients who did not receive any nutrition during ECMO course, as listed in order by time on ECMO.

Patient	1° dx	2° dx	3° dx	Age	Gender	ECMO	ECMO Indication	Reason for Delay h
7	Streptococcal septicemia			0–2	M	<24	Streptococcal septic shock (cardiac)	Patient expired within 24 h of ECMO
10	Tracheitis			0–2	M	<24	Acute chronic respiratory failure which progressed to cardiac arrest (non cardiac)	Patient expired within 24 h of ECMO
19	Pulmonary Insufficiency/ Shock Lung	ARDS and ALI	Acute Hypoxic Respiratory Failure	2–6	F	<24	Cardiac arrest (noncardiac)	Expired within 24 h of admission
20	Pulmonary Insufficiency/ Shock Lung	ARDS and ALI	Acute Hypoxic Respiratory Failure	0–2	M	<24	Cardiac arrest (cardiac)	Less than 48 h timeframe
21	Cardiogenic Shock			0–2	F	<24	Respiratory Failure (noncardiac)	Expired within 24 h of starting ECMO
22	Conduction Disorders of Heart	Interference/ Atrioventricular Dissociation		>12	M	<24	Cardiogenic Shock (Cardiac)	Patient expired within 24 h of starting ECMO
153	Multi-organ failure in the presence of *E.coli* bacteremia			0–2	F	<24	Lactic acidosis (noncardiac)	Patient expired within 24 h of ECMO
4	Meconium aspiration	Respiratory failure	Sepsis	0–2	F	24–48	Meconium aspiration syndrome of newborn (noncardiac)	Less than 48 h timeframe
150	Cardiac Arrest			0–2	F	24–48	Cardiac arrest (cardiac)	GI bleed and unable to obtain access for TPN/lipids
156	Atresia of esophagus with trachea-esophageal fistula			0–2	M	48–72	Cardiac arrest (cardiac)	Fluid restriction s/p surgery and holding feeds due to attempted extubation and subsequent reintubation and initiation of ECMO
165	Cardiac arrest			>12	M	48–72	Cardiac arrest (cardiac)	Fluid overload, kidney status, and clinical status; Patient expired prior to initiating feeds
169	Truncus arteriosus communis			0–2	M	48–72	Heart failure (Cardiac)	Fluid overload (diastolic heart failure, needed peritoneal drains)
143	Cardiovascular failure secondary to congenital heart disease surgery			0–2	M	72–96	Cardiac Failure (Cardiac)	Fluid status
1	Tetralogy of Fallot	Imperforate Anus		0–2	M	>96	Shock bowel (noncardiac)	Procedures GI intolerance (abdominal catastrophe and abdominal decompression) Clinical status (septic shock)
18	Influenza virus infection			2–6	M	>96	Acute hypoxic respiratory failure (noncardiac)	Fluid overload
144	Sepsis			>12	F	>96	Cardiac shock secondary to COVID-19 (cardiac)	Fluid overload

Notes: Age in years. ALI: Acute lung injury; ARDS: acute respiratory distress syndrome; ECMO: Extracorporeal membrane oxygenation; GI: gastrointestinal; TPN: total parenteral nutrition.

**Table 2:** A summary of the dosages of vasoactive inotrope drugs and vasoactive inotrope scores (N = 583) in the PICU over 467 ECMO days.

Drug	Units	(N)	Missing	Unique	Min	Max	Mean	StDev	Sum	0.25	0.5	0.75	0.95
Dopamine	mcg/kg/min	581	5 (0.9%)	9	0	10	0.42	1.47	244	0	0	0	5
Dobutamine	mcg/kg/min	581	5 (0.9%)	2	0	2	0	0.08	2	0	0	0	0
Epinephrine	mcg/kg/min	582	4 (0.7%)	39	0	1.5	0.04	0.12	20.5	0	0	0.04	0.1
Milrinone	mcg/kg/min	582	4 (0.7%)	16	0	0.75	0.29	0.26	167.29	0	0.3	0.5	0.75
Vasopressin	U/kg/min	582	4 (0.7%)	25	0	0.07	0	0.01	0.67	0	0	0	0.01
Norepinephrine	mcg/kg/min	582	4 (0.7%)	27	0	1.55	0.01	0.07	8.04	0	0	0	0.06
Vasoactive Inotrope Score	N/A	582	4 (0.7%)	93	0	780	19.68	61.08	11,453.90	2	6	10.5	105.2

**Table 3:** Linear regression—outcome, total calories.

Variable	DF	Parameter Estimate	*t* Value	Pr > |*t*|	Variance Inflation	95% Confidence Limits	
Intercept	1	335.56065	2.97	0.0031	0	113.55558	557.56573
ECMO_pump	1	−305.33290	−7.72	<0.0001	1.69695	−383.03861	−227.62718
ECMO_type	1	422.06974	9.03	<0.0001	1.72180	330.29957	513.83991
VIS_total_calc	1	−0.54393	−2.44	0.0150	1.01924	−0.98171	−0.10614

Notes: Dependent variable: total_calories; number of observations read: 600; number of observations used: 584; number of observations with missing values: 16.

**Table 4:** Linear regression—outcome, total proteins.

Variable	DF	Parameter Estimate	*t* Value	Pr > |*t*|	Variance Inflation	95% Confidence Limits	
Intercept	1	18.28493	2.11	0.0350	0	1.29131	35.27854
ECMO_pump	1	−24.47442	−8.10	<0.0001	1.69228	−30.40675	−18.54208
ECMO_type	1	33.01087	9.32	<0.0001	1.69287	26.05432	39.96742
ECMO_comp	1	6.18347	2.98	0.0030	1.00060	2.10472	10.26222

Notes: Dependent variable: total_protein; number of observations read: 600; number of observations used: 584; number of observations with missing values: 16.

**Table 5:** Interruption of nutritional support.

Variable	Overall Patients (*n* = 64)
Nutritional Support Interruptions	
Yes	12 (18.8)
No	35 (54.7)
N/A	17 (26.6)
Number of Interruptions	*n* = 12
1	11 (91.7)
6	1 (8.3)

Notes: Data are reported as count (%).

**Table 6:** Delayed nutritional support descriptions.

Group	Delayed NS (N = 12)	No Delayed NS (N = 36)	*p*-Value
Age Group			0.7280 *
Neonatal	3 (20.0)	12 (80.0)	
Pediatric	9 (27.3)	24 (72.7)	
ECMO Type			1.0000 *
VA	10 (25.6)	29 (74.4)	
VV	2 (22.2)	7 (77.8)	
ECMO Pump Type			1.0000 *
Centrifugal	4 (26.7)	11 (73.3)	
Roller	8 (24.2)	25 (75.8)	
ECMO Indication			0.0665
Cardiac	9 (36.0)	16 (64.0)	
Non-Cardiac	3 (13.0)	20 (87.0)	
Previous Cardiac Arres			0.3156
Yes	7 (31.8)	15 (68.2)	
No	5 (19.2)	21 (80.8)	
ECMO Complications			0.4042
Yes	7 (30.4)	16 (69.6)	
No	5 (20.0)	20 (80.0)	
Median VIS	8 [5, 10]	6 [0, 9]	0.3549

Notes: Abbreviations: NS: Nutritional support. Numeric data were expressed as median [25th, 75th percentile] and analyzed using Wilcoxon Rank Sum. Categorical analyses were completed using Chi-Square or Fisher’s exact test (denoted with * on the *p*-value) and data reported as count (%). The percentages were reported at the row level instead of within the delayed category fields. We removed the 16 N/A delayed feeds individuals so we could accurately assess those who had observations for delayed feeds.

## Data Availability

The original contributions presented in the study are included in the article/Supplementary Material, further inquiries can be directed to the corresponding author.

## Supplementary Materials

The following supporting information can be downloaded at: https://www.mdpi.com/article/10.3390/nu16223813/s1, Supplementary Figure S1: Scatterplots of inotrope (dopamine, dobutamine, epinephrine, milirinone, vasopressin, norepinephrin) dosages (*n* = 583) in PICU over 467 ECMO days; Supplementary Table S1: Heatmap and raw data of vasopressor dosages over consecutive days on ECMO; Supplementary Table S2: VIS by sub-category and analysis; Supplementary Table S3: Correlations: VIS vs. calories/protein by independent variables; Supplementary Table S4: Feed interruptions by categories (N = 47).

## Abbreviations

ALI: Acute lung injury; ARDS: acute respiratory distress syndrome; ASPEN: American Society of Parenteral and Enteral Nutrition; CRRT: continuous renal replacement therapy; ECMO: extracorporeal membrane oxygenation; ELSO: Extracorporeal Life Support Organization; GI: gastrointestinal; NIH: National Institute of Health; PCICU: Pediatric Cardiac Intensive Care Unit; PICU: pediatric intensive care unit; SCCM: Society of Critical Care Medicine; VA ECMO: venoarterial; VIS: vasoactive ionotropic score; VV ECMO: venovenous.
