# Supplementary material for: Nutritional Intake in Venovenous ECMO Patients: A Single-Center Study in a North American PICU
Source: Nutrients. 2024 Nov 7;16(22):3813. doi: 10.3390/nu16223813 (PMC11597126; doi:10.3390/nu16223813)
Supplement: Supplementary file 1 [file nutrients-16-03813-s001.zip › nutrients-3279411-supplementary.pdf]

Supplemental Figure S1: Scatterplots of Ionotropes (Dopamine, Dobutamine, Epinephrine, Milirinone, Vasopressin, Norepinephrin) Dosages (n=583; Y-axis) in PICU over 467 ECMO days.

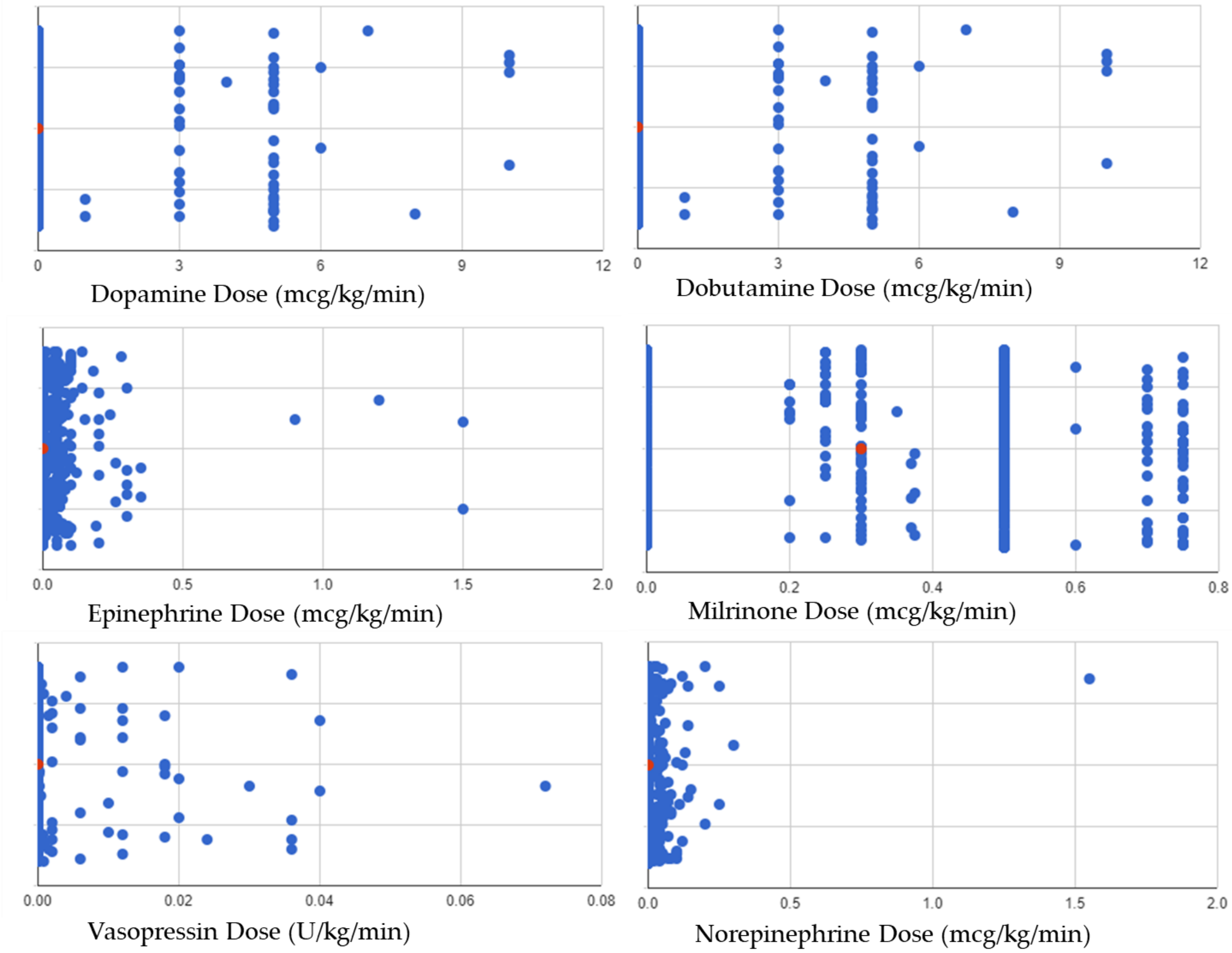

Supplemental Table S1: Heatmap and Raw Data of Vasopressor Dosages over Consecutive days on ECMO

record_ id	repeat_ instance	dopa_dose	dobut_ dose	epi_ dose	milrin_ dose	vasopres_ dose	norepi_ dose	vis_total_ calc
**1**	1	0	0	0.01	0.7	0	0.02	10
**1**	2	0	0	0.03	0.3	0	0.07	13
**1**	3	0	0	0.03	0.3	0	0.12	18
**1**	4	0	0	0.02	0.5	0	0	7
**1**	5	0	0	0	0.5	0	0	5
**1**	6	0	0	0	0	0	0.14	14
**2**	1	0	0	0.05	0.3	0	0	8
**2**	2	0	0	0.06	0.5	0	0	11
**2**	3	0	0	0.05	0.3	0	0	8
**2**	4	0	0	0.05	0.3	0	0.02	10
**2**	5	0	0	0.05	0.3	0	0	8
**2**	6	0	0	0.1	0.3	0	0	13
**3**	1	0	0	0	0	0	0	0
**3**	2	0	0	0	0	0	0	0
**3**	3	0	0	0	0	0	0	0
**3**	4	0	0	0	0	0	0	0
**3**	5	0	0	0	0	0	0	0
**3**	6	0	0	0	0	0	0	0
**4**	1	3	0	0	0.5	0	0	8
**4**	2	5	0	0	0.5	0	0	10
**4**	3	5	0	0	0.5	0	0	10
**5**	1	3	0	0	0.5	0	0	8
**5**	2	0	0	0	0.75	0	0	7.5
**5**	3	0	0	0	0.75	0	0	7.5
**5**	4	0	0	0	0.75	0	0	7.5
**5**	5	0	0	0	0.75	0	0	7.5
**5**	6	0	0	0	0.5	0	0	5
**5**	7	0	0	0	0.5	0	0	5
**5**	8	0	0	0	0.3	0	0	3
**5**	9	0	0	0	0.5	0	0	5
**5**	10	0	0	0	0.5	0	0	5
**5**	11	0	0	0	0.5	0	0	5
**5**	12	0	0	0	0.5	0	0	5
**5**	13	0	0	0	0.5	0	0	5
**5**	14	0	0	0	0.5	0	0	5
**5**	15	0	0	0	0	0	0	0
**6**	1	0	0	0	0.3	0	0	3
**6**	2	0	0	0	0.75	0	0	7.5
**6**	3	0	0	0	0.75	0	0	7.5

**6**	4	0	0	0	0.75	0	0	7.5
**6**	5	0	0	0	0.75	0	0	7.5
**6**	6	0	0	0	0.75	0	0	7.5
**6**	7	0	0	0	0.75	0	0	7.5
**6**	8	0	0	0	0.75	0	0	7.5
**6**	9	0	0	0	0.5	0	0	5
**6**	10	0	0	0	0.5	0	0	5
**6**	11	0	0	0	0.3	0	0	3
**6**	12	0	0	0	0.3	0	0	3
**6**	13	0	0	0	0.3	0	0	3
**6**	14	0	0	0	0.3	0	0	3
**7**	1	0	0	0.12	0.5	0	0	17
**7**	2	0	0	0.2	0.5	0	0	25
**8**	1	0	0	0	0	0	0.02	2
**8**	2	0	0	0	0	0	0	0
**9**	1	0	0	0.11	0.25	0.04	0.03	416.5
**9**	2	0	0	0.03	0.25	0.04	0.04	409.5
**9**	3	0	0	0.03	0.25	0.02	0.03	208.5
**9**	4	0	0	0.01	0.25	0.01	0.03	106.5
**9**	5	0	0	0.07	0.25	0	0.05	14.5
**10**	1	0	0	0.03	0.5	0	0	8
**10**	2	0	0	0.2	0.3	0	0	23
**11**	1	0	0	0.06	0.5	0	0	11
**11**	2	0	0	0.04	0.5	0	0	9
**11**	3	0	0	0.03	0.5	0	0	8
**11**	4	0	0	0.03	0.5	0	0	8
**11**	5	0	0	0.03	0.5	0	0	8
**11**	6	0	0	0.05	0.5	0	0	10
**11**	7	0	0	0.05	0.5	0	0	10
**11**	8	0	0	0.09	0.5	0	0	14
**11**	9	0	0	0.07	0.5	0	0	12
**11**	10	0	0	0.05	0.5	0	0	10
**11**	11	0	0	0.06	0.5	0	0	11
**11**	12	0	0	0.03	0.5	0	0	8
**11**	13	0	0	0	0.5	0	0	5
**11**	14	0	0	0	0.5	0	0	5
**11**	15	0	0	0.02	0.5	0	0	7
**11**	16	0	0	0.03	0.5	0	0	8
**11**	17	0	0	0.03	0.5	0	0	8
**11**	18	0	0	0.03	0.5	0	0	8
**11**	19	0	0	0.01	0.5	0	0	6
**11**	20	0	0	0.05	0.5	0	0	10
**12**	8	0	0	0	0	0	0	0

**12**	9	0	0	0	0	0	0	0
**12**	10	0	0	0	0	0	0	0
**12**	11	0	0	0	0	0	0	0
**12**	12	0	0	0	0	0	0	0
**12**	13	0	0	0	0	0	0	0
**12**	14	0	0	0	0	0	0	0
**12**	15	0	0	0	0	0	0	0
**13**	1	0	0	0	0.5	0	0	5
**13**	2	5	0	0	0.5	0	0	10
**13**	3	4	0	0	0.5	0	0	9
**13**	4	5	0	0	0.5	0	0	10
**13**	5	3	0	0	0.5	0	0	8
**13**	6	3	0	0	0.5	0	0	8
**13**	7	0	0	0	0.5	0	0	5
**13**	8	0	0	0	0.5	0	0	5
**13**	9	5	0	0	0.5	0	0	10
**13**	10	8	0	0	0.5	0	0	13
**13**	11	5	0	0	0.5	0	0	10
**13**	12	3	0	0	0.5	0	0	8
**13**	13	5	0	0	0.5	0	0	10
**13**	14	5	0	0	0.5	0	0	10
**13**	15	3	0	0	0.5	0	0	8
**13**	16	3	0	0	0.5	0	0	8
**13**	17	6	0	0	0.5	0	0	11
**13**	18	6	0	0.1	0.5	6	0	81
**14**	1	0	0	0.05	0.75	0.02	0	212.5
**14**	2	0	0	0.05	0.75	0.012	0	132.5
**14**	3	0	0	0	0.5	0	0	5
**14**	4	0	0	0	0.5	0	0	5
**14**	5	5	0	0.05	0.5	0	0	15
**15**	1	5	0	0.02	0.25	0.02	0	209.5
**15**	2	5	0	0	0.5	0.01	0	110
**15**	3	3	0	0	0.5	0	0	8
**15**	4	3	0	0	0.5	0	0	8
**15**	5	3	0	0	0.5	0	0	8
**15**	6	3	0	0	0.5	0	0	8
**15**	7	3	0	0	0.5	0	0	8
**15**	8	3	0	0	0.5	0	0	8
**16**	1	0	0	0.01	0	0	0	1
**16**	2	0	0	0	0	0	0	0
**16**	3	0	0	0	0	0	0	0
**16**	4	0	0	0	0	0	0	0
**16**	5	0	0	0	0	0	0	0

**16**	6	0	0	0	0	0	0	0
**16**	7	0	0	0	0	0	0	0
**17**	1	0	0	0.35	0	0	0	35
**17**	2	0	0	0	0	0	0	0
**17**	3	0	0	0	0	0	0	0
**17**	4	0	0	0	0	0	0	0
**17**	5	0	0	0	0	0	0	0
**17**	6	0	0	0	0	0	0	0
**17**	7	0	0	0	0	0	0	0
**17**	8	0	0	0	0	0	0	0
**17**	9	0	0	0	0	0	0	0
**17**	10	0	0	0	0	0	0	0
**17**	11	0	0	0	0	0	0.02	2
**17**	12	0	0	0	0	0	0	0
**18**	1	0	0	0	0	0	0	0
**18**	2	0	0	0	0	0	0	0
**18**	3	0	0	0	0	0	0	0
**18**	4	0	0	0	0	0	0	0
**18**	5	0	0	0	0	0	0	0
**18**	6	0	0	0	0.5	0	0	5
**19**	1	0	0	0.3	0	0	0	30
**20**	1	0	0	0	0	0	0.03	3
**20**	2	0	0	0.07	0.3	0	0.07	17
**21**	1	0	0	0	0.5	0	0	5
**21**	2	0	0	0	0	0	0	0
**22**	1	0	0	0.26	0.25	0	0	28.5
**22**	2	0	0	0	0	0	0	0
**23**	1	0	0	0.3	0	0.0008	0	38
**23**	2	0	0	0.3	0.2	0	0	32
**23**	3	0	0	0	0	0	0	0
**24**	1	0	0	0.03	0.7	0	0	10
**24**	2	0	0	0.03	0.7	0	0	10
**24**	3	0	0	0.03	0.7	0	0	10
**24**	4	0	0	0.05	0.6	0	0	11
**25**	1	0	0	0.04	0	0	0	4
**25**	2	0	0	0	0	0	0	0
**25**	3	0	0	0	0	0	0	0
**25**	4	0	0	0	0	0	0	0
**26**	1	0	0	0.04	0.5	0	0	9
**26**	2	0	0	0.06	0	0	0	6
**26**	3	0	0	0.06	0.5	0	0	11
**26**	4	0	0	0.06	0.5	0	0	11
**26**	5	0	0	1.5	0.25	0	0.05	157.5

**27**	1	10	0	0.2	0.5	0	0.02	37
**27**	2	10	0	0.05	0.5	0	0	20
**27**	3	5	0	0	0.5	0	0	10
**27**	4	0	0	0	0.5	0	0	5
**27**	5	0	0	0	0	0	0	0
**28**	1	0	0	0	0.5	0	0	5
**28**	2	0	0	0	0	0	0	0
**28**	3	5	0	0	0.5	0	0	10
**28**	4	0	0	0.08	0.5	0	0	13
**28**	5	0	0	0.1	0.5	0	0	15
**28**	6	0	0	0	0	0	0	0
**29**	1	0	0	0.14	0.5	0	0	19
**29**	2	0	0	0.09	0.5	0	0	14
**29**	3	0	0	0.09	0.5	0	0	14
**29**	4	0	0	0.07	0.7	0	0	14
**29**	5	0	0	0.09	0.7	0	0	16
**29**	6	0	0	0.2	0.7	0	0	27
**30**	1	0	0	0.05	0.5	0	0	10
**30**	2	0	0	0	0.5	0	0	5
**30**	3	0	0	0	0.5	0	0	5
**30**	4	0	0	0.02	0.5	0	0	7
**30**	5	0	0	0.02	0.5	0	0	7
**30**	6	0	0	0.07	0.5	0	0	12
**30**	7	0	2	0.03	0.7	0	0	12
**31**	1	0	0	0.07	0.5	0	0.05	17
**31**	2	0	0	0.05	0.5	0	0.03	13
**31**	3	0	0	0.04	0.7	0	0.02	13
**31**	4	0	0	0.04	0.7	0	0	11
**31**	5	0	0	0.04	0.7	0	0	11
**31**	6	0	0	0.04	0.7	0	0	11
**31**	7	0	0	0.04	0.7	0	0	11
**31**	8	0	0	0.07	0.5	0	0	12
**32**	1	0	0	0	0	0	0	0
**32**	2	0	0	0	0	0	0	0
**32**	3	0	0	0	0	0	0	0
**32**	4	0	0	0	0	0	0	0
**32**	5	0	0	0	0	0	0	0
**32**	6	0	0	0	0	0	0	0
**32**	7	0	0	0	0	0	0	0
**32**	8	0	0	0	0	0	0	0
**33**	1	0	0	0	0	0	0	0
**33**	2	0	0	0.08	0.25	0	0	10.5
**33**	3	0	0	0.03	0	0	0	3

**33**	4	0	0	0	0	0	0	0
**33**	5	0	0	0	0	0	0	0
**33**	6	0	0	0.1	0.35	0	0	13.5
**33**	7	0	0	0.1	0.25	0	0	12.5
**33**	8	0	0	0.1	0.25	0	0	12.5
**33**	9	0	0	1.2	0	0	0	120
**34**	1	5	0	0.1	0.3	0	0.05	23
**34**	2	0	0	0.1	0.3	0	0.05	18
**34**	3	0	0	0.08	0.3	0	0.04	15
**34**	4	0	0	0.1	0	0	0.05	15
**34**	5	0	0	0.06	0.3	0	0.04	13
**34**	6	0	0	0.06	0.3	0	0.04	13
**34**	7	0	0	0.06	0.3	0	0.04	13
**34**	8	0	0	0.1	0.3	0	0.04	17
**34**	9	0	0	0.06	0.3	0	0.02	11
**34**	10	0	0	0.07	0	0	0.07	14
**142**	1	0	0	0.18	0	0.002	0.12	50
**142**	2	0	0	0	0.2	0	0.04	6
**142**	3	0	0	0	0.5	0	0	5
**142**	4	0	0	0	0.5	0	0	5
**143**	1	0	0	0	0.75	0	0	7.5
**143**	2	0	0	0.03	0.75	0	0	10.5
**143**	3	0	0	0.03	0.75	0	0	10.5
**143**	4	0	0	0.05	0.75	0	0	12.5
**144**	1	0	0	1.5	0.5	0.0015	1.55	325
**144**	2	0	0	0.9	0.5	0	0.14	109
**144**	3	0	0	0.35	0.5	0	0	40
**144**	4	0	0	0.28	0.5	0	0	33
**144**	5	0	0	0.26	0.5	0	0	31
**145**	1	0	0	0	0.7	0	0	7
**145**	2	0	0	0.07	0.5	0	0	12
**145**	3	0	0	0.05	0.5	0	0	10
**145**	4	0	0	0	0.5	0	0	5
**145**	5	0	0	0	0.5	0	0	5
**145**	6	0	0	0	0.5	0	0	5
**145**	7	0	0	0	0.5	0	0	5
**146**	1	0	0	0.05	0.5	0	0	10
**146**	2	0	0	0.05	0.5	0	0	10
**146**	3	0	0	0.05	0.5	0	0	10
**146**	4	0	0	0	0.5	0	0	5
**146**	5	0	0	0.02	0.5	0	0	7
**146**	6	0	0	0	0.5	0	0	5
**146**	7	0	0	0	0.5	0	0	5

**146**	8	0	0	0	0.5	0	0	5
**146**	9	0	0	0	0.5	0	0	5
**146**	10	0	0	0	0.5	0	0	5
**146**	11	0	0	0	0.5	0	0	5
**146**	12	0	0	0	0.5	0	0	5
**146**	13	0	0	0	0.5	0	0	5
**146**	14	0	0	0	0.5	0	0	5
**146**	15	0	0	0	0.5	0	0	5
**146**	16	0	0	0	0.5	0	0	5
**146**	17	0	0	0	0	0	0	0
**146**	18	0	0	0	0	0	0	0
**146**	19	0	0	0.06	0	0	0	6
**146**	20	0	0	0.06	0	0	0.14	20
**146**	21	0	0	0.06	0	0	0.08	14
**146**	22	0	0	0.06	0	0	0.08	14
**146**	23	0	0	0.06	0	0	0	6
**146**	24	0	0	0.06	0	0	0	6
**146**	25	0	0	0.04	0	0	0.04	8
**146**	26	0	0	0.04	0	0	0	4
**146**	27	0	0	0.04	0	0	0	4
**146**	28	0	0	0.04	0	0	0.01	5
**146**	29	0	0	0.04	0	0	0.04	8
**146**	30	0	0	0	0	0	0.05	5
**146**	31	0	0	0	0	0	0.1	10
**146**	32	0	0	0	0	0	0.08	8
**146**	33	0	0	0	0	0	0.08	8
**147**	1	0	0	0.24	0.5	0	0	29
**147**	2	0	0	0.05	0.5	0	0	10
**147**	3	0	0	0	0.5	0	0	5
**147**	4	0	0	0	0.5	0	0	5
**148**	1	0	0	0	0.5	0	0.06	11
**148**	2	0	0	0	0.5	0	0.05	10
**148**	3	0	0	0	0.7	0	0	7
**148**	4	0	0	0	0.5	0	0	5
**148**	5	0	0	0	0.5	0	0	5
**148**	6	0	0	0	0.5	0	0.02	7
**148**	7	0	0	0	0.5	0	0.05	10
**148**	8	0	0	0	0.3	0	0.07	10
**148**	9	0	0	0	0.3	0	0.05	8
**148**	10	0	0	0	0	0	0.05	5
**148**	11	0	0	0	0	0	0.05	5
**148**	12	0	0	0	0	0.0004	0	4
**148**	13	0	0	0	0	0.0008	0	8

**148**	14	0	0	0	0	0.0008	0	8
**148**	15	0	0	0	0	0.0015	0.13	28
**149**	1	7	0	0	0.5	0	0	12
**149**	2	5	0	0	0.25	0	0	7.5
**149**	3	0	0	0	0.5	0	0	5
**149**	4	0	0	0	0.5	0	0	5
**149**	5	0	0	0	0.5	0	0	5
**149**	6	0	0	0	0.5	0	0	5
**149**	7	0	0	0	0.5	0.002	0	25
**150**	1	0	0	0.09	0	0	0	9
**150**	2	0	0	0.08	0.2	0	0	10
**150**	3	0	0	0.03	0	0	0.03	6
**151**	1	0	0	0	0	0.0004	0	4
**151**	2	0	0	0	0.75	0	0	7.5
**151**	3	0	0	0	0.75	0	0	7.5
**151**	4	0	0	0	0.75	0	0	7.5
**151**	5	0	0	0	0.75	0	0	7.5
**151**	6	0	0	0	0.5	0	0	5
**152**	1	0	0	0	0.3	0.0002	0.25	30
**152**	2	0	0	0	0.3	0	0.25	28
**152**	3	0	0	0	0.3	0	0	3
**152**	4	0	0	0	0.3	0	0	3
**152**	5	0	0	0	0.5	0	0	5
**152**	6	0	0	0	0.5	0	0	5
**152**	7	0	0	0	0.3	0	0.03	6
**152**	8	0	0	0	0.3	0	0.03	6
**152**	9	0	0	0	0.5	0.00015	0.15	21.5
**153**	1	0	0	0.3	0	0	0.2	50
**155**	1	0	0	0	0	0	0	0
**155**	2	0	0	0	0	0	0	0
**155**	3	0	0	0	0	0	0	0
**155**	4	0	0	0	0	0	0	0
**155**	5	0	0	0	0	0	0	0
**155**	6	0	0	0	0	0	0	0
**155**	7	0	0	0	0	0	0	0
**155**	8	0	0	0	0	0	0.03	3
**155**	9	0	0	0	0	0.002	0.06	26
**155**	10	0	0	0	0	0	0.02	2
**155**	11	0	0	0	0	0	0	0
**155**	12	0	0	0	0	0	0	0
**155**	13	0	0	0	0	0	0.01	1
**155**	14	0	0	0	0	0	0	0
**155**	15	0	0	0	0	0	0	0

**155**	16	0	0	0	0	0	0	0
**155**	17	0	0	0	0	0	0	0
**155**	18	0	0	0	0	0	0	0
**155**	19	0	0	0	0	0	0	0
**155**	20	0	0	0	0	0	0	0
**155**	21	0	0	0	0	0	0	0
**155**	22	0	0	0	0	0	0	0
**155**	23	0	0	0	0	0	0	0
**155**	24	0	0	0	0	0	0	0
**155**	25	0	0	0	0	0.002	0	20
**155**	26	0	0	0	0	0.004	0.03	43
**155**	27	0	0	0	0	0.002	0.02	22
**155**	28	0	0	0	0	0.002	0.02	22
**155**	29	0	0	0	0	0.002	0.01	21
**155**	30	0	0	0	0	0.002	0.02	22
**155**	31	0	0	0	0	0	0.06	6
**155**	32	0	0	0	0	0	0.06	6
**155**	33	0	0	0	0	0	0.04	4
**155**	34	0	0	0	0	0	0.04	4
**155**	35	0	0	0	0	0	0.06	6
**155**	36	0	0	0	0	0	0.03	3
**155**	37	0	0	0	0	0	0.04	4
**155**	38	0	0	0	0	0	0.08	8
**155**	39	0	0	0	0	0	0.02	2
**155**	40	0	0	0	0	0	0	0
**155**	41	0	0	0	0	0	0.01	1
**155**	42	0	0	0	0	0	0.01	1
**155**	43	0	0	0	0	0	0	0
**156**	1	0	0	0	0.7	0	0	7
**156**	2	0	0	0.04	0.7	0	0	11
**156**	3	0	0	0	0	0	0	0
**157**	1	5	0	0.1	0.25	0.036	0	377.5
**157**	2	5	0	0.1	0.5	0.036	0	380
**157**	3	5	0	0.1	0.5	0	0	20
**157**	4	5	0	0.1	0.5	0	0	20
**157**	5	5	0	0.08	0.5	0	0	18
**157**	6	5	0	0.08	0.75	0.012	0	140.5
**157**	7	10	0	0.08	0.75	0	0	25.5
**157**	8	3	0	0.08	0.75	0.012	0	138.5
**157**	9	3	0	0.06	0.75	0.012	0	136.5
**157**	10	0	0	0	0.75	0.006	0	67.5
**157**	11	0	0	0.06	0.75	0	0	13.5
**157**	12	0	0	0.06	0.75	0	0	13.5

**157**	13	0	0	0.03	0.75	0	0	10.5
**157**	14	0	0	0.03	0.75	0	0	10.5
**157**	15	0	0	0.03	0.5	0.03	0	308
**157**	16	0	0	0.03	0.3	0.018	0	186
**157**	17	0	0	0.03	0.3	0.012	0	126
**157**	18	0	0	0.03	0.3	0	0	6
**157**	19	0	0	0	0.5	0	0	5
**157**	20	0	0	0	0.5	0	0	5
**157**	21	0	0	0	0.5	0	0	5
**157**	22	0	0	0	0.5	0	0	5
**157**	23	0	0	0	0.5	0	0	5
**157**	24	0	0	0	0.5	0	0	5
**157**	25	0	0	0	0.5	0	0	5
**157**	26	0	0	0	0	0	0	0
**157**	27	0	0	0	0.5	0	0	5
**157**	28	0	0	0	0.5	0	0	5
**157**	29	0	0	0	0.5	0	0	5
**157**	30	0	0	0	0.5	0	0	5
**157**	31	0	0	0	0.5	0	0	5
**157**	32	0	0	0	0.5	0	0	5
**157**	33	0	0	0	0.5	0	0	5
**157**	34	0	0	0	0.5	0	0	5
**157**	35	0	0	0	0.5	0	0	5
**157**	36	0	0	0	0.5	0	0	5
**157**	37	0	0	0	0.5	0	0	5
**157**	38	0	0	0	0.5	0	0	5
**157**	39	5	0	0	0	0	0	5
**157**	40	1	0	0	0.5	0.006	0	66
**157**	41	1	0	0	0.5	0.006	0	66
**157**	42	5	0	0	0.5	0.012	0	130
**157**	43	5	0	0	0.5	0.006	0	70
**158**	1	0	0	0	0	0	0	0
**158**	2	0	0	0	0	0	0	0
**158**	3	0	0	0	0	0	0	0
**158**	4	0	0	0	0	0	0	0
**158**	5	0	0	0	0	0	0	0
**158**	6	0	0	0	0	0	0	0
**158**	7	0	0	0	0	0	0	0
**158**	8	0	0	0	0	0	0	0
**158**	9	0	0	0	0	0	0	0
**158**	10	0	0	0	0	0	0	0
**158**	11	0	0	0	0	0	0	0
**159**	1	0	0	0.01	0.25	0	0	3.5

**159**	2	0	0	0.015	0	0	0	1.5
**159**	3	0	0	0.04	0.25	0	0	6.5
**159**	4	0	0	0.04	0.25	0	0	6.5
**159**	5	0	0	0	0.5	0.018	0	185
**159**	6	0	0	0	0.5	0.018	0	185
**159**	7	0	0	0	0.3	0	0.05	8
**159**	8	0	0	0	0.3	0.012	0.06	129
**159**	9	0	0	0	0	0.006	0.02	62
**159**	10	0	0	0	0.3	0.018	0.03	186
**159**	11	0	0	0	0	0	0	0
**159**	12	0	0	0	0	0	0	0
**159**	13	0	0	0.08	0	0.018	0.04	192
**160**	1	0	0	0.15	0	0	0	15
**160**	2	0	0	0.03	0	0	0	3
**160**	3	0	0	0.03	0.5	0	0	8
**160**	4	0	0	0.03	0.5	0	0	8
**160**	5	0	0	0.03	0.5	0	0	8
**160**	6	0	0	0.03	0.3	0	0	6
**160**	7	0	0	0.07	0	0	0	7
**160**	8	0	0	0.14	0	0	0	14
**160**	9	0	0	0.2	0	0	0	20
**161**	1	0	0	0.05	0	0	0	5
**161**	2	0	0	0	0	0	0	0
**161**	3	0	0	0	0	0	0	0
**161**	4	0	0	0	0	0	0	0
**161**	5	0	0	0	0	0	0	0
**161**	6	0	0	0	0	0	0	0
**161**	7	0	0	0	0	0	0	0
**161**	8	0	0	0	0	0	0	0
**161**	9	0	0	0	0	0	0	0
**161**	10	0	0	0	0	0	0	0
**161**	11	0	0	0	0	0	0	0
**161**	12	0	0	0	0	0	0	0
**161**	13	0	0	0	0	0	0	0
**161**	14	0	0	0	0	0	0	0
**161**	15	0	0	0	0	0	0	0
**162**	1	10	0	0	0.5	0.024	0	255
**162**	2	3	0	0	0.5	0.036	0	368
**162**	3	3	0	0	0.5	0	0	8
**162**	4	0	0	0	0.25	0	0	2.5
**162**	5	0	0	0	0.5	0	0	5
**162**	6	0	0	0	0.5	0	0	5

**162**	7	0	0	0	0.5	0	0	5
**163**	1	0	0	0.02	0	0	0.02	4
**163**	2	0	0	0	0	0	0	0
**163**	3	0	0	0	0	0	0	0
**163**	4	0	0	0	0	0	0	0
**163**	5	0	0	0	0	0	0	0
**163**	6	0	0	0	0.5	0	0	5
**163**	7	0	0	0	0.5	0	0	5
**163**	8	0	0	0.05	0.5	0	0	10
**163**	9	0	0	0	0.5	0	0	5
**163**	10	0	0	0.05	0.5	0	0	10
**163**	11	0	0	0	0.25	0	0	2.5
**163**	12	0	0	0.01	0	0	0	1
**164**	1	0	0	0	0	0	0	0
**164**	2	0	0	0	0	0	0	0
**164**	3	0	0	0	0	0	0	0
**164**	4	0	0	0	0	0	0	0
**164**	5	0	0	0	0	0	0	0
**165**	1	0	0	0.1	0	0	0	10
**165**	2	0	0	0.2	0.25	0.036	0.2	402.5
**165**	3	0	0	0.3	0	0.072	0.3	780
**165**	4	0	0	0	0	0	0	0
**166**	1	0	0	0	0	0	0.12	12
**166**	2	0	0	0	0	0	0	0
**166**	3	0	0	0	0	0	0	0
**166**	4	0	0	0	0	0	0	0
**166**	5	0	0	0	0	0	0	0
**167**	1	0	0	0.05	0.5	0	0	10
**167**	2	0	0	0.05	0.5	0	0.02	12
**167**	3	0	0	0.05	0.6	0	0	11
**167**	4	0	0	0.05	0.5	0	0.02	12
**167**	5	0	0	0.05	0.3	0	0	8
**167**	6	0	0	0.19	0.75	0	0.05	31.5
**167**	7	0	0	0.05	0.5	0	0.05	15
**167**	8	0	0	0.06	0.5	0	0	11
**167**	9	0	0	0.04	0.5	0	0	9
**167**	10	0	0	0.06	0	0	0	6
**167**	11	0	0	0.05	0.5	0	0.04	14
**168**	1	0	0	0.05	0.3	0	0	8
**168**	2	0	0	0.06	0.3	0	0	9
**168**	3	0	0	0.05	0.75	0	0	12.5
**168**	4	0	0	0.05	0.5	0	0	10
**168**	5	0	0	0.03	0.5	0	0	8

**168**	6	0	0	0.05	0.5	0	0	10
**168**	7	0	0	0.03	0.5	0	0	8
**168**	8	0	0	0.03	0.5	0	0	8
**168**	9	0	0	0.01	0.5	0	0	6
**169**	1	0	0	0	0.75	0	0	7.5
**169**	2	0	0	0	0.75	0	0	7.5
**169**	3	0	0	0	0	0	0	0
**170**	1	0	0	0.05	0.5	0	0.05	15
**170**	2	0	0	0.02	0	0.0002	0	4
**170**	3	0	0	0.02	0	0	0	2
**170**	4	0	0	0.02	0	0	0	2
**170**	5	0	0	0	0	0	0	0
**170**	6	0	0	0	0.2	0	0	2
**170**	7	0	0	0	0.5	0	0	5
**170**	8	0	0	0	0.37	0	0.03	6.7
**170**	9	0	0	0	0.37	0	0.04	7.7
**170**	10	0	0	0	0.375	0	0.01	4.75
**170**	11	0	0	0	0.375	0	0.03	6.75
**170**	12	0	0	0	0.37	0	0	3.7
**170**	13	0	0	0	0.375	0	0.03	6.75
**171**	1	0	0	0	0.5	0	0	5
**171**	2	0	0	0	0.75	0	0	7.5
**171**	3	0	0	0	0.75	0	0	7.5
**171**	4	0	0	0.04	0.6	0	0	10
**171**	5	0	0	0	0.5	0	0	5
**171**	6	0	0	0	0.3	0	0	3
**171**	7	0	0	0	0	0	0	0
**171**	8	0	0	0	0	0	0	0
**171**	9	0	0	0	0	0	0	0
**171**	10	0	0	0	0	0	0	0
**171**	11	0	0	0	0	0	0.07	7
**171**	12	0	0	0	0	0	0.06	6
**171**	13	0	0	0	0	0	0.02	2
**171**	14	0	0	0.05	0.5	0	0	10
**171**	15	0	0	0.05	0.3	0	0.04	12
**171**	16	0	0	0.05	0.3	0	0	8
**171**	17	0	0	0.05	0.3	0	0	8
**171**	18	0	0	0.05	0.3	0	0	8
**171**	19	0	0	0.05	0.3	0	0	8
**171**	20	0	0	0.05	0.3	0	0	8
**171**	21	0	0	0.07	0.3	0	0	10
**171**	22	0	0	0.07	0.5	0	0	12
**171**	23	0	0	0	0.7	0	0	7

**171**	24	0	0	0	0	0	0	0
**172**	1	0	0	0.03	0	0	0	3
**172**	2	0	0	0.03	0	0	0	3
**172**	3	0	0	0.03	0	0	0	3
**172**	4	0	0	0.06	0	0	0	6
**172**	5	0	0	0.08	0	0	0	8
**172**	6	0	0	0.05	0	0	0.1	15
**172**	7	0	0	0.05	0	0	0.1	15
**172**	8	0	0	0.09	0	0	0.1	19
**172**	9	0	0	0.05	0	0	0.05	10
**172**	10	0	0	0.1	0.25	0.0007	0.11	30.5
**172**	11	0	0	0	0	0.0005	0.03	8
**172**	12	0	0	0	0	0.0002	0.02	4
**172**	13	0	0	0	0	0	0	0
**172**	14	0	0	0.03	0.2	0	0.05	10
**172**	15	0	0	0.03	0.2	0	0.05	10
**172**	16	0	0	0.03	0.2	0	0.04	9
**172**	17	0	0	0.03	0.2	0	0	5
**172**	18	0	0	0.03	0	0	0	3
**172**	19	0	0	0.05	0	0	0	5
**172**	20	0	0	0.06	0	0	0	6
**172**	21	0	0	0.06	0	0	0	6
**172**	22	0	0	0	0	0	0	0
**173**	1	0	0	0.03	0	0	0	3
**173**	2	0	0	0	0	0	0	0
**173**	3	0	0	0	0	0	0	0
**173**	4	0	0	0	0	0	0	0
**173**	5	0	0	0	0	0	0	0
**173**	6	0	0	0	0	0	0	0
	Legend	0	0	0	0	0	0	0
		1-3	1-3	0.01-0.5	0.1-0.25	0.0001-0.01	0.01-0.3	0-6
		4-7		0.6-1.0	0.26-0.5	0.011-1.0	0.4-1.0	7-12
		8-10		1.1-1.5	0.51-0.75	1.0-10.0	1.1-2.0	12-24
								>24
								Mean= 19.68
								Median=6

Supplemental Table S2: VIS by sub-category and analysis

Total VIS Score by Cardiac Status

- Comparing total VIS score across ECMO indication (cardiac vs non-cardiac) using a Wilcoxon Rank Sum test.

Group	Cardiac (N = 285)	Non-cardiac (N = 308)	P-value
VIS total	8.0 [5.0, 12.0]	4.0 [0.0, 8.0]	<.0001

- There is statistically significant evidence to suggest a difference in VIS score between ECMO indication groups. VIS score is higher among those in the cardiac group (8.0 [5.0, 12.0]) compared to those in the non-cardiac group (4.0 [0.0, 8.0]) (P <.0001).

Total VIS Score by Age Group

- Comparing total VIS score across age group using a Wilcoxon Rank Sum test.

Group	Neonatal N = 216	Pediatric N =377	P-value
VIS total	7.5 [5.0, 10.3]	5.0 [0.0, 11.0]	<.0001

- There is statistically significant evidence to suggest a difference in VIS score between age groups. VIS score is higher among those in the neonatal group (7.5 [5.0, 10.3]) compared to those in the pediatric group (5.0 [0.0, 11.0]) (P <.0001).

Total VIS Score by ECMO Type

- Comparing total VIS score across ECMO type using a Wilcoxon Rank Sum test.

Group	VA N = 489	VV N = 104	P-value
VIS total	7.5 [5.0, 11.0]	0.0 [0.0, 1.5]	<.0001

- There is statistically significant evidence to suggest a difference in VIS score between ECMO type groups. VIS score is higher among those in the VA group (7.5 [5.0, 11.0]) compared to those in the VV group (0.0 [0.0, 1.5]) (P <.0001).

Total VIS Score by ECMO Pump Type

- Comparing total VIS score across ECMO pump type using a Wilcoxon Rank Sum test.

Group	Centrifugal	Roller	P-value

	N = 163	N =430	
VIS total	2.0 [0.0, 8.0]	7.5 [4.0, 11.0]	<.0001

- There is statistically significant evidence to suggest a difference in VIS score between ECMO pump groups. VIS score is higher among those in the roller group (7.5 [4.0, 11.0]) compared to those in the centrifugal group (2.0 [0.0, 8.0]) (P <.0001).

Total VIS Score by Previous Cardiac Arrest

- Comparing total VIS score across history of cardiac arrest using a Wilcoxon Rank Sum test.

Group	Yes<br>N = 230	No<br>N =363	P-value
VIS total	8.0 [5.0, 12.0]	5.0 [0.0, 10.0]	<.0001

- There is statistically significant evidence to suggest a difference in VIS score between previous cardiac arrest groups. VIS score is higher among those who had cardiac arrest (8.0 [5.0, 12.0]) compared to those who did not (5.0 [0.0, 10.0]) (P <.0001).

Total VIS Score by ECMO Complications

- Comparing total VIS score across ECMO complication groups using a Wilcoxon Rank Sum test.

Group	Yes<br>N = 306	No<br>N = 287	P-value
VIS total	7.5 [3.0, 11.0]	5.0 [0.0, 10.0]	0.0013

- There is statistically significant evidence to suggest a difference in VIS score between ECMO complication groups. VIS score is higher among those who had complications (7.5 [3.0, 11.0]) compared to those who did not (5.0 [0.0, 10.0]) (P = 0.0013).

Supplemental Table S3: Correlations: VIS Score vs. Calories/Protein by Independent Variables

	ECMO Complications – Yes		ECMO Complications – No	
	**VIS Correlation Coefficient (N=301)**	***p*-value**	**VIS Correlation Coefficient (N=283)**	***p*-value**
**Total Calories**	-0.12904	**0.0252**	-0.15415	**0.0094**
**Total Protein**	-0.10818	0.0609	-0.11257	0.0586

	ECMO Indication - Cardiac		ECMO Indication Non-cardiac	
	**VIS Correlation Coefficient (N=278)**	***p*-value**	**VIS Correlation Coefficient (N=306)**	***p*-value**
**Total Calories**	-0.12981	**0.0305**	-0.14649	**0.0103**
**Total Protein**	-0.11598	0.0534	-0.11046	0.0536

	Age Group - Neonatal		Age Group - Pediatric	
	**VIS Correlation Coefficient (N=213)**	***p*-value**	**VIS Correlation Coefficient (N=371)**	***p*-value**
**Total Calories**	-0.14694	**0.0321**	-0.14649	**0.0047**
**Total Protein**	-0.10509	0.1263	-0.11782	**0.0232**

	ECMO Type - VA		ECMO Type - VV	
	**VIS Correlation Coefficient (N=481)**	***p*-value**	**VIS Correlation Coefficient (N=103)**	***p*-value**
**Total Calories**	-0.12784	**0.0050**	0.02502	0.8019
**Total Protein**	-0.09578	**0.0357**	0.12083	0.2241

	ECMO Pump Type - Centrifugal		ECMO Pump Type - Roller	
	**VIS Correlation Coefficient (N=161)**	***p*-value**	**VIS Correlation Coefficient (N=423)**	***p*-value**
**Total Calories**	-0.17335	**0.0279**	-0.14868	**0.0022**
**Total Protein**	-0.15234	0.0537	-0.08481	0.0815

Cardiac arrest 24 hours prior to ECMO -Yes vs. - No

	**VIS Correlation Coefficient (N=225)**	***p*-value**	**VIS Correlation Coefficient (N=359)**	***p*-value**
**Total Calories**	-0.10198	0.1272	-0.16263	**0.0020**
**Total Protein**	-0.09675	0.1480	-0.12365	**0.0191**

Supplemental Table S4 – Feed Interruptions by Categories (N=47)

Group	Feed Interruptions (N=12)	No Feed Interruptions (N=35)	*p*-value
Age Group			
Neonatal	3 (20.0)	12 (80.0)	0.7252*
Pediatric	9 (28.1)	23 (71.9)	
ECMO Type			
VA	11 (29.0)	27 (71.0)	0.4122*
VV	1 (11.1)	8 (88.9)	
ECMO Pump Type			
Centrifugal	2 (14.3)	12 (85.7)	0.3017*
Roller	10 (30.3)	23 (69.7)	
ECMO Indication			
Cardiac	7 (29.2)	17 (70.8)	0.5594
Non-Cardiac	5 (21.7)	18 (78.3)	
Previous Cardiac Arrest			
Yes	7 (33.3)	14 (66.7)	0.2703
No	5 (19.2)	21 (80.8)	
ECMO Complications			
Yes	7 (31.8)	15 (68.2)	0.3538
No	5 (20.0)	20 (80.0)	
Median VIS Score	8 [3, 9]	6 [0, 10]	0.8437

Notes: Categorical analyses were completed using Chi-Square or Fishers exact test (denoted with * on the p-value) and data reported as count (percent). The percentages were reported at the row level instead of the within the delayed category fields. Numeric data was expressed as median [25th, 75th percentile] and analyzed using Wilcoxon Rank Sum.
